# HisCoM-PCA: software for hierarchical structural component analysis for pathway analysis based using principal component analysis

**DOI:** 10.5808/GI.2020.18.1.e11

**Published:** 2020-03-31

**Authors:** Nan Jiang, Sungyoung Lee, Taesung Park

**Affiliations:** 1 Interdisciplinary Program in Bioinformatics, Seoul National University, Seoul 08826, Korea; 2 Center for Precision Medicine, Seoul National University Hospital, Seoul 08826, Korea; 3 Department of Statistics, Seoul National University, Seoul 08826, Korea

**Keywords:** genome-wide association study, hierarchical structural component model, pathway analysis, principal component analysis

## Abstract

In genome-wide association studies, pathway-based analysis has been widely performed to enhance interpretation of single-nucleotide polymorphism association results. We proposed a novel method of hierarchical structural component model (HisCoM) for pathway analysis of common variants (HisCoM for pathway analysis of common variants [HisCoM-PCA]) which was used to identify pathways associated with traits. HisCoM-PCA is based on principal component analysis (PCA) for dimensional reduction of single nucleotide polymorphisms in each gene, and the HisCoM for pathway analysis. In this study, we developed a HisCoM-PCA software for the hierarchical pathway analysis of common variants. HisCoM-PCA software has several features. Various principle component scores selection criteria in PCA step can be specified by users who want to summarize common variants at each gene-level by different threshold values. In addition, multiple public pathway databases and customized pathway information can be used to perform pathway analysis. We expect that HisCoM-PCA software will be useful for users to perform powerful pathway analysis.

**Availability:** HisCoM-PCA is available on the website (http://statgen.snu.ac.kr/software/HisCom-PCA/index.html).

## Introduction

In genome-wide association studies (GWAS), researchers have identified many single-nucleotide polymorphisms (SNPs) associated with the traits of interest (phenotypes) [1]. However, these SNPs sometimes may sometimes suffer from a lack of biological interpretation [2]. To enhance interpretation of SNP association results, many gene-based analysis and pathway-based analysis have been widely performed in GWAS. For examples, PHARAOH was developed for pathway analysis of rare variants, and hierarchical structural component analysis of gene-gene interactions (HisCoM-GGI) was proposed for gene-gene interaction analysis of common variants [3,4].

Recently, we presented a hierarchical structural component model (HisCoM) for pathway analysis of common variants (HisCoM-PCA) to identify pathways associated with traits [5]. HisCoM-PCA is based on principal component analysis (PCA) for dimension reduction of SNPs in each gene, and the HisCoM for pathway analysis. In the dimensional reduction step of HisCoM-PCA, various principle component scores (PC) selection criteria may be used. The criterion may be defined by a threshold of cumulative proportion of variances. It can also be defined by using only the first PC for each gene. In the pathway analysis step, multiple published pathway databases and specific combination of pathways can be used to identify pathways associated with the traits of interest.

In our previous study, we used only pathway information of the Kyoto Encyclopedia of Genes and Genome pathway database [6] and adopted two criteria to select PC: (1) using only the first PC and (2) using the PCs whose cumulative proportion of variances are more than 30%. To enable researchers to perform various pathway analysis, we developed HisCoM-PCA software for allowing the users to set the PC selection criterion and pathway information flexibly.

## Implementation

**Fig. 1.**
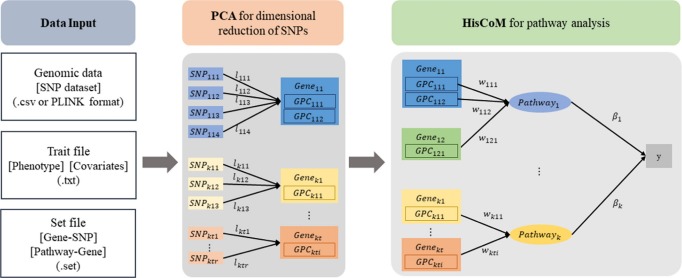
The workflow of the HisCoM-PCA software. PCA, principal component analysis; SNP, single nucleotide polymorphism; HisCoM-PCA, hierarchical structural component model for pathway analysis of common variants.

The workflow of the HisCoM-PCA software is shown in Fig. 1. The HisCoM-PCA mehod has been proposed for pathway analysis of common variants by constructing a hierarchical model using SNP-gene-pathway information. The HisCoM-PCA method consists of two steps: (1) dimensional reduction of SNPs by PCA and (2) pathway analysis with a hierarchical component model. In the first dimensional reduction step of HisCoM-PCA software, the user can define the number of PCs for each gene by one of the following two options: (1) the threshold of cumulative proportion of variances and (2) only the first PC. Using the selected PCs, pathway analysis can be performed based on the user-entered pathway datasets. In the second pathway analysis step, HisCoM-PCA utilizes ridge-type penalization and performs a permutation test to estimate the gene and pathway effects on the phenotypes.

### Input file

The HisCoM-PCA software takes four inputs: (1) a SNP dataset in csv file or PLINK format files, (2) a txt file with phenotype and covariate(s), (3) a set file that consists of two columns for gene name and SNP id, and (4) a set file that consists of two columns for pathway name and gene name. Furthermore, the program also accepts the published pathway databases in MsigDB [7,8].

### Output file

The HisCoM-PCA program generates the following output files: (1) a ‘[prefix]. gene-pca_summary.csv’ file that contains the number of SNPs and PCs for each gene, (2) a ‘[prefix].gene.ressum.csv’ file that contains pathway name, gene name, number of permutation, weight of gene for the pathway, gene coefficient, and permutation p-value of gene, and (3) a ’[prefix].pathway.ressum.csv’ file that contains pathway name, number of permutation, number of genes in each pathway, pathway coefficient, and permutation p-value of pathway.

## Conclusion

We introduced our HisCoM-PCA software for pathway analysis of common variants in GWAS. HisCoM-PCA software supports pathway analyses using multiple candidate pathways and customized PC selection criterion. We expect that our HisCoM-PCA software is useful for users to perform various pathway analyses. This section should contain sufficient detail so that all procedures can be repeated, in conjunction with the cited references. The manufacturer and model number should be stated in this section—for example, as Sigma Chemical Co. (St. Louis, MO, USA).
